# Genetic resources for methane production from biomass described with the Gene Ontology

**DOI:** 10.3389/fmicb.2014.00634

**Published:** 2014-12-03

**Authors:** Endang Purwantini, Trudy Torto-Alalibo, Jane Lomax, João C. Setubal, Brett M. Tyler, Biswarup Mukhopadhyay

**Affiliations:** ^1^Department of Biochemistry, Virginia Polytechnic Institute and State UniversityBlacksburg, VA, USA; ^2^European Bioinformatics Institute (EMBL-EBI), European Molecular Biology LaboratoryHinxton, UK; ^3^Department of Biochemistry, Universidade de São PauloSão Paulo, Brazil; ^4^Virginia Bioinformatics Institute, Virginia Polytechnic Institute and State UniversityBlacksburg, VA, USA; ^5^Center for Genome Research and Biocomputing, Oregon State UniversityCorvallis, OR, USA; ^6^Department of Biological Sciences, Virginia Polytechnic Institute and State UniversityBlacksburg, VA, USA

**Keywords:** Gene Ontology, biomass, biodegradation, methanogenesis, methanogen, bioenergy, carbon cycle, waste treatment

## Abstract

Methane (CH_4_) is a valuable fuel, constituting 70–95% of natural gas, and a potent greenhouse gas. Release of CH_4_ into the atmosphere contributes to climate change. Biological CH_4_ production or methanogenesis is mostly performed by methanogens, a group of strictly anaerobic archaea. The direct substrates for methanogenesis are H_2_ plus CO_2_, acetate, formate, methylamines, methanol, methyl sulfides, and ethanol or a secondary alcohol plus CO_2_. In numerous anaerobic niches in nature, methanogenesis facilitates mineralization of complex biopolymers such as carbohydrates, lipids and proteins generated by primary producers. Thus, methanogens are critical players in the global carbon cycle. The same process is used in anaerobic treatment of municipal, industrial and agricultural wastes, reducing the biological pollutants in the wastes and generating methane. It also holds potential for commercial production of natural gas from renewable resources. This process operates in digestive systems of many animals, including cattle, and humans. In contrast, in deep-sea hydrothermal vents methanogenesis is a primary production process, allowing chemosynthesis of biomaterials from H_2_ plus CO_2_. In this report we present Gene Ontology (GO) terms that can be used to describe processes, functions and cellular components involved in methanogenic biodegradation and biosynthesis of specialized coenzymes that methanogens use. Some of these GO terms were previously available and the rest were generated in our Microbial Energy Gene Ontology (MENGO) project. A recently discovered non-canonical CH_4_ production process is also described. We have performed manual GO annotation of selected methanogenesis genes, based on experimental evidence, providing “gold standards” for machine annotation and automated discovery of methanogenesis genes or systems in diverse genomes. Most of the GO-related information presented in this report is available at the MENGO website (http://www.mengo.biochem.vt.edu/).

## Introduction

**Figure 1:**
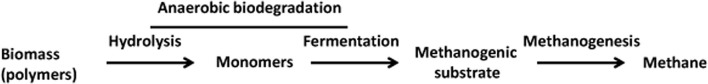
**Methanogenic degradation of biomass—an overview**. Examples: Polymers—cellulose, hemicellulose, and proteins; Monomers: glucose, xylose, and amino acids. Methanogenic substrates: hydrogen plus carbon dioxide, formate, acetate, and methanol.

Methane (CH_4_), the simplest aliphatic hydrocarbon, is a valuable fuel. It constitutes 70–95% (volume/volume) of natural gas (Strapoc et al., 2011). The biological production of methane, which occurs under strictly anaerobic conditions, is critical to the operation of the global carbon cycle, nutrient recovery in the digestive systems of numerous animals, and treatment of municipal and industrial wastes, and it could potentially allow commercial production of methane from renewable resources (Zinder, 1993; Thauer et al., 2008; McInerney et al., 2009). The methane present in geological deposits such as oil and gas reservoirs and coal beds also originated in part from microbial degradation of biomass, and the rest of it was derived from thermal maturation of the remnants from biodegradation (Strapoc et al., 2011). Each of these cases involves anaerobic degradation of biopolymers such as carbohydrates and proteins, as well as lipids, and this process is composed of two broad steps (Figure 1): first, generation of substrates for methanogens through a combination of hydrolysis and fermentation; second, methanogenesis or methane production. Methanogenesis is also one of the most ancient respiratory processes on Earth, developing 2.7–3.2 billion years ago, and by virtue of the processes described above it continues to be an important process on the present day Earth (Leigh, 2002). Furthermore, biological methanogenesis is a significant contributor to climate change as together with water vapor, carbon dioxide and ozone, methane also contributes to the greenhouse effect (Strapoc et al., 2011). According to United States Environmental Protection Agency (US EPA) “Pound for pound, the comparative impact of CH_4_ on climate change is over 20 times greater than CO_2_ over a 100-year period” (http://epa.gov/climatechange/ghgemissions/gases/ch4.html).

For the ecological, evolutionary, and applied interests discussed in the preceding paragraph, methanogens have been investigated intensely in the past six decades (Wolfe, 1991; Thauer, 1998, 2012). This research has resulted into a detailed understanding of the biochemistry of these archaea, especially their unique energy metabolism, methanogenesis, and the mechanistic details of their interactions with other microorganisms in numerous ecological niches. For the same reasons, genomes of methanogens have been analyzed from the early days of genome sequencing. In fact, *Methanocaldococcus jannaschii*, a methanogen, was the first archaeon and third organism to be targeted for complete genome sequence determination (Bult et al., 1996). Since then the genome sequences of more than 170 methanogens have appeared in public databases. These genomes have not only helped to advance the research on methanogens, but also have catalyzed major shifts in our understanding of the relationships of these organisms with the rest of the biological world. It is now known that many of the biological parts and processes that were once thought to be specific to methanogenic archaea are major contributors to the metabolism of numerous non-methanogenic organisms from all domains of life (Takao et al., 1989; Batschauer, 1993; Purwantini et al., 1997; Graham and White, 2002; Chistoserdova et al., 2004; Krishnakumar et al., 2008). Often such discoveries have been based on the detection of methanogen genes in non-methanogen genomes, followed by biochemical analysis of their molecular functions and knowledge based deductions of their roles in the metabolic pathways in those organisms. In this context a rich set of GO terms fully describing methanogenesis together with manually generated gene annotations based on experimental evidence (gold standards) could bring great strength, as it would provide expanded qualifications of the methanogen genes in a non-methanogen genome, such as predicted functions and cellular locations of the gene products, through automated analysis. This resource will then allow facile mining of useful parts of methanogenesis systems from both methanogens as well as non-methanogenic organisms. The Gene Ontology (GO) consists of three sets of terms for describing gene products in terms of biological processes (GO:0008150), cellular components (GO:0005575), and molecular functions (GO:0003674) (Ashburner et al., 2000). These terms are related to each other in a semi-hierarchical fashion (a directed graph structure), from very broad terms (at the top of the hierarchy) to specific (at the bottom). GO annotation can thus provide both specific and broader attributes to gene products. This is the primary motivation for the work described in this report.

## Gene ontology (GO) describing methanogenesis

The promise cited above has inspired the work on the methanogenesis component of our MENGO (Microbial Energy production Gene Ontology) project. The goal of the MENGO project is to develop a set of GO terms for describing gene products involved in energy-related microbial processes. A major focus is on the microbial biomass degradation for the production of biofuel (fuel from renewable resources) such as methane, alcohols, fatty acid esters, hydrocarbons, and hydrogen. Until now we have generated 667 terms and these are available at the GO website (AMIGO: http://tinyurl.com/kh7fqne) as well as at our MENGO website (http://www.mengo.biochem.vt.edu/). Of these, 563 terms are in the Biological Process ontology, 88 in the Molecular Function ontology and 16 in the Cellular Component ontology. More terms are still under review (GO:MENGO-UR) and when the respective GO ID's are assigned, we will post those at the MENGO website (http://www.mengo.biochem.vt.edu/). We generated these terms in two ways: 1. Our own effort, which involved a review of the relevant literature and creation of terms as the needs were identified. 2. Community input, where MENGO terms were generated following suggestions from the members of the bioenergy research community who attended the MENGO workshops organized by us at the following locations: Great Lakes Bioenergy Research Center, University of Wisconsin, Madison, WI (2011); Annual User Meeting of the US Department of Energy Joint Genome Institute, Walnut Creek, CA (2011 and 2012); US Department of Energy's Genomic Science Awardee Meeting IX and X (Crystal City, VA, and Bethesda, MD, respectively) (2011 and 2012).

In this report we present GO terms suitable for describing processes, functions and cellular components involved in methanogenic biodegradation of biomass, including methanogenesis, in the context of both natural and engineered processes. We begin this description with a brief review of the relevant systems. More detailed information, especially the mechanistic details of methanogenesis, is available in several reviews including some cited here (Wolfe, 1993; Zinder, 1993; Ferry, 1999; Deppenmeier, 2002; Liu and Whitman, 2008; Thauer et al., 2008; Thauer, 2012; Costa and Leigh, 2014; Welte and Deppenmeier, 2014). Furthermore, to remain focused on bioconversion or catabolism, the general cellular biosynthesis processes have not been covered in this report; exceptions are the syntheses of coenzymes that were once thought to be unique to methanogens (Wolfe, 1991, 1992; Graham and White, 2002) and afterwards some of these were found to occur in the bacteria (Purwantini et al., 1997; Chistoserdova et al., 2004; Krishnakumar et al., 2008). Recently, a non-canonical route that contributes significantly to global biological production of methane has been described (Metcalf et al., 2012) and we describe this system briefly. In numerous environments, complete anaerobic biodegradation of biomass can occur without the formation of methane and here processes such as sulfate reduction and acetogenesis provide avenues for the disposal of reductants (Isa et al., 1986; Gibson et al., 1988; Widdel, 1988; Zinder, 1993; Breznak, 1994; De Graeve et al., 1994; Raskin et al., 1996; Muller, 2003). Those processes will not be covered here.

The work on the GO for methanogenesis began with a review of the GO database. This showed that, although the GO terms describing many of the biological processes and molecular functions associated with methane biosynthesis were available, the coverage of this area was incomplete. To fill this gap we generated an additional 110 GO terms for methanogenesis. A comprehensive source of this information is on our website (http://www.mengo.biochem.vt.edu/) where the data are available under two menus: MENGO (All MENGO Terms; Process Specific; Ontology Specific; New MENGO Terms) and PATHWAYS (Natural Pathways; Synthetic/Engineered Pathways). Under the MENGO menu a form (Submit MENGO Term) is available for the submission of new terms that will help to describe gene products involved in methanogenesis in a comprehensive manner and to validate the resource through research community input.

## Methanogenic degradation of biomass

As mentioned in the Introduction, this process is composed of two broad steps, anaerobic biodegradation of biomass generating substrates for methanogens, and methanogenesis (Figure 1). The narrative appearing below covers both natural and engineered systems.

### Anaerobic biodegradation of biomass

#### Natural systems

**Figure 2:**
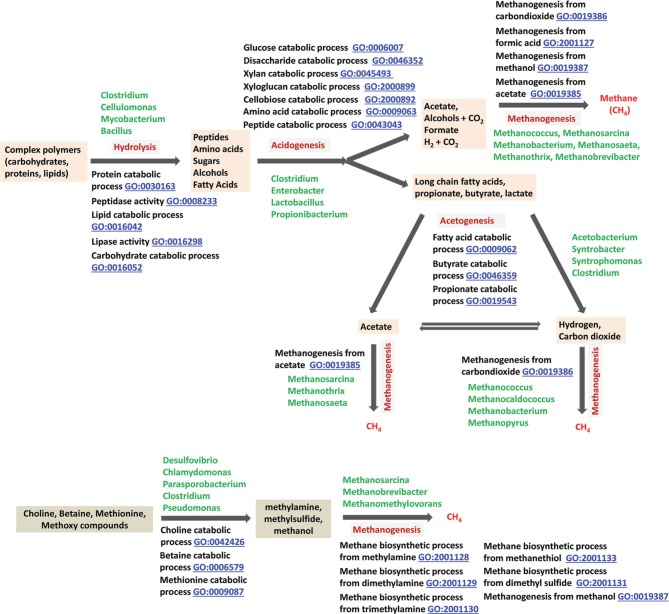
**Anaerobic digestion of complex biopolymers to methane, and relevant GO terms**. The degradation of large polymers found in biomass starts with their hydrolytic fragmentation to monomers by biological hydrolysis (Zinder, 1993); in industry, chemical processes are often used for this deconstruction step (Blanch et al., 2011). Monomers are degraded further to methanogenic substrates first by acidogenic and then by acetogenic microorganisms (Zinder, 1993). Finally, methanogenic archaea transform these methanogenic substrates such as formate, hydrogen, carbon dioxide, acetate, methylamines and methanol to methane (Zinder, 1993). Methylamines originate from choline and betaine by the actions of choline/betaine degrading microorganisms. Microbial degradation of pectin is a common source of methanol in nature (Schink et al., 1981).

***Anaerobic biodegradation of biomass in sediments***. Annually, plant and photosynthetic microorganisms fix 70 billion tons of carbon into biomass made up of complex biopolymers, such as cellulose, hemicellulose, lignin, proteins and lipids (Thauer et al., 2008). About 1% of this material is mineralized in various anaerobic niches of nature through a process that yields methane and carbon dioxide as end products (Figure 2). The combination of photosynthesis (GO:0015979) and macromolecule catabolism (GO:0009057) constitutes the biological component of the biogeochemical process of carbon cycling.

Cellulose is a polymer of D-glucose units connected by β (1→4) bonds. The anaerobic mineralization of cellulose (synonym of “cellulose catabolic process, anaerobic,” GO:1990488) starts with hydrolysis of the β (1→4) bonds by cellulases (GO:0008810) produced by anaerobic cellulolytic bacteria and fungi (Adney et al., 1991; Teunissen and Op Den Camp, 1993; Leschine, 1995; Li et al., 1997; Schwarz, 2001; Ljungdahl, 2008; Ransom-Jones et al., 2012). These organisms either secrete the cellulases or carry these enzymes on their cell surfaces (Teunissen and Op Den Camp, 1993; Li et al., 1997). A recent study shows that excreted enzymes with multiple catalytic sites and multiple cellulose-binding modules provide *Caldicellulosiruptor bescii*, an anaerobic thermophile with a high activity of cellulose degradation (Brunecky et al., 2013). The cellulose catabolic process (GO:0030245) involves the actions of endo-β-1,4-glucanases (GO:0052859) and exo-1,4-β-glucanases or cellobiohydrolases (CBH) (reducing-end-specific, GO:0033945; non-reducing-end-specific, GO:0016162) that generate cellobiose, with intermediate formation of fragments with multiple glucose units (Akin, 1980; Beguin and Aubert, 1994; Bayer et al., 1998; Perez et al., 2002; Hilden and Johansson, 2004), and hydrolysis of cellobiose to glucose (cellobiose catabolic process, GO:2000892) by β-glucosidase (GO:0008422).

**Figure 3:**
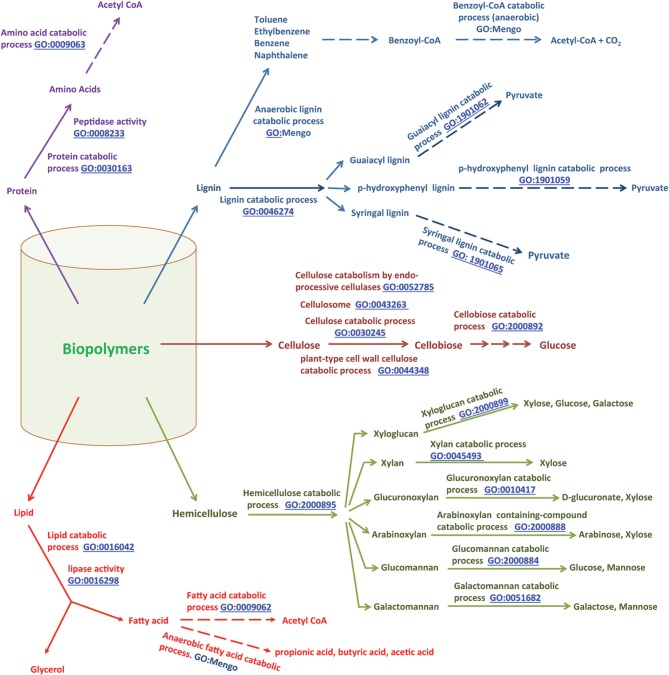
**General pathways for biopolymer degradation, and relevant GO terms**. Biopolymers such as cellulose, hemicellulose, lipids, proteins and lignin are converted to their respective monomers/oligomers. Monomers are further catabolized to simple compounds which then can be metabolized by microorganisms to generate useful materials, such as renewable biofuel. The relevant references are in the text. This is a modified version of a figure available at our MENGO project website: http://www.mengo.biochem.vt.edu/pathways/bio_synthetic_pathways.php.

The cellulose degrading anaerobic microorganisms and other non-cellulolytic anaerobes with access to the products generated by cellulolytic microbes take up and ferment D-glucose to acetate, alcohols, lactate and fatty acids (e.g., propionate, butyrate) via respective biosynthetic processes (Figure 3) (Zinder, 1993; Schink, 1997; Ahring, 2003; McInerney et al., 2009). The butyrate biosynthetic process (GO:0046358) involves an intermediate formation of acetyl-CoA (acetyl-CoA biosynthetic process, GO:0006085) whereas for propionate biosynthesis (GO:0019542) succinate generated via the tricarboxylic acid metabolic process or TCA cycle (GO:0072350) serves as the direct precursor (Zinder, 1993; Schink, 1997; Ahring, 2003; McInerney et al., 2009).

Butyrate and propionate, which are called short-chain fatty acids (SCFAs), are further fermented to acetate, hydrogen and CO_2_ (fatty acid catabolic process: GO:0009062; child term, anaerobic fatty acid catabolic process, GO:1990486) via their respective catabolic processes (butyrate catabolic process, GO:0046359; propionate catabolic process, GO:0019543) (Zinder, 1993; Schink, 1997; Ahring, 2003; McInerney et al., 2009); *Syntrophobacter, Syntrophomonas, Syntrophus, Smithella*, and *Pelotomaculum* species are some of the bacteria that produce these SCFAs. Ethanol and lactate are also fermented to acetate, hydrogen and CO_2_ (ethanol catabolic process, GO:0006068; anaerobic lactic acid catabolic, process GO:1990485). The hydrogen biosynthetic process (GO:1902422) is a key element of these fermentation processes and those described in the preceding paragraph for the following reason. Several steps of fermentation lead to the reduction of electron carriers such as NAD^+^ and ferredoxin, producing NADH and reduced-ferredoxin. For the fermentation process to continue, NAD^+^ and ferredoxin must be regenerated, and often the only available route to meet this requirement is the reduction of protons, yielding molecular hydrogen (H_2_) (GO:1902422) (Zinder, 1993; Schink, 1997; Ahring, 2003; McInerney et al., 2009).

Degradation of hemicellulose follows a path similar to that described for cellulose (summarized in Figure 3). The term hemicellulose includes xylan (polymer of xylose), glucuronoxylan (polymer of D-glucuronate and xylose), arabinoxylan (polymer of arabinose and xylose), glucomannan (polymer of glucose and mannose), galactomannan (polymer of galactose and mannose), and xyloglucan (polymer of xylose, glucose and galactose) (Akin, 1980; Perez et al., 2002). These are degraded via specific hemicellulose catabolic processes (GO:2000895) to their respective monomers (Akin, 1980; Perez et al., 2002). The fermentation of monomers yields acetate, hydrogen and CO_2_ (Wolin and Miller, 1983; Schink, 1997).

Lignin degradation in anaerobic environments (anaerobic lignin catabolic process, GO:1990487) is not well studied and is considered rare to impossible (Akin, 1980; Harwood and Gibson, 1988; Perez et al., 2002; Fuchs, 2008); the broader lignin catabolic process is generally considered an aerobic process (Perez et al., 2002). However, following the degradation of lignin by aerobic microorganisms such as fungi, a variety of aromatic compounds (catechol, benzoate, p-hydroxybenzoate, vanillate-, ferulate, syringate, p-hydroxybenzoate, p-hydroxycinnamate, and 3-methoxy-4-hydroxyphenylpyruvate) (Kaiser and Hanselmann, 1982a,b) become available in anaerobic environments. Fermentation of these aromatic compounds by anaerobic bacteria leads to acetate, CO_2_ and hydrogen (Harwood and Parales, 1996; Fuchs, 2008). Anaerobic degradation of benzoate, one of the lignin monomers, has been studied in detail and this catabolic process (benzoate catabolic process via CoA ligation, GO:0010128) yields acetate and CO_2_ (Figure 3). The metabolism of several other lignin monomers by anaerobes has also been investigated (Harwood and Parales, 1996; Fuchs, 2008) and some of the relevant information for vanillin, ferulate and catechol is summarized in Figure 3.

Anaerobic lipid catabolic processes also lead to acetate and hydrogen (McInerney, 1988; Zinder, 1993; Schink, 1997). The process begins with the hydrolysis of lipids (lipase activity, GO:0016298); the broader lipid catabolic process is represented by GO:0016042. The glycerol released by hydrolysis enters the glycolysis pathway generating acetate and hydrogen (Zinder, 1993) (Figure 3). The fatty acid units are degraded via the β-oxidation pathway (fatty acid beta-oxidation, GO:0006635) to acetate and the excess reducing equivalents are released as hydrogen (Figure 3). In the case of proteins, the amino acids released by the action of proteases or peptidases (peptidase activity, GO:0008233; endopeptidase activity GO:0004175, exopeptidase activity, GO:0008238) are deaminated oxidatively, releasing ammonia and hydrogen, and then the resulting ketoacids are fermented to acetate and hydrogen (McInerney, 1988; Zinder, 1993; Schink, 1997) (Figure 3).

In each of the above cases, as H_2_ accumulates the oxidation of reduced electron carriers becomes thermodynamically unfavorable and consequently the fermentation process slows down or even halts (McInerney, 1988; Zinder, 1993; Schink, 1997). Methanogens consume hydrogen and reduce CO_2_ to methane, thus relieving the block on fermentation (McInerney, 1988; Zinder, 1993; Schink, 1997). These archaea also convert acetate to methane and CO_2_ and this action also improves the thermodynamics of biodegradation (Zinder, 1993). As CH_4_ moves to aerobic zones, such as the surface of water overlaying sediments, methanotrophic bacteria oxidize this hydrocarbon to CO_2_ (methane catabolic process, GO:0046188) (Kiene, 1991; Zinder, 1993; Conrad, 2007, 2009). More recent work shows that significant amount of methane is oxidized anaerobically and the microbial basis and mechanistic details of this process are beginning to emerge (Conrad, 2009; Thauer, 2011; Milucka et al., 2012; Shima et al., 2012; Haroon et al., 2013; Offre et al., 2013). Hence, by removing the hydrogen-induced thermodynamic block and converting acetate to methane, methanogens facilitate the complete degradation of the biopolymers discussed above.

In marine anaerobic sediments rich in sulfates some of the products of biomass degradation also lead to methane production. In general however, in this environment hydrogen and acetate are not available to the methanogens as, in the presence of sulfate, sulfate-reducing bacteria readily use these materials to reduce sulfate to hydrogen sulfide (dissimilatory sulfate reduction, GO:0019420), and the growth rates and affinities for H_2_ of the sulfate-reducing bacteria are much higher than those of the methanogens (Widdel, 1988). However, several hydrogen-consuming methanogens belonging to the class of Methanococci have been isolated from marine environments (Whitman et al., 1986). It has been speculated that these organisms may depend primarily on formate which could arise from the catabolism of oxalate (GO:0033611) derived from plant materials (Allison et al., 1985); most methanococci are capable of consuming both hydrogen and formate (Boone et al., 1993).

A significant amount of methane is also produced from methylamines, methylsulfides and carbon monoxide (Zinder, 1993; Thauer, 1998; Deppenmeier et al., 1999; Ferry, 1999, 2011; Liu and Whitman, 2008; Thauer et al., 2008). The sources of methylamines are betaine and choline, (GO:0006579 and GO:0042426, respectively) while methylsulfides are generated from sulfur-containing compounds such as methionine and dimethylpropiothetin (GO:0009087; and GO:0047869, respectively; Figure 3) (Boone et al., 1993). In certain marine environments, carbon monoxide provided by kelp algae provides both reductant and carbon for methanogenesis (methane biosynthetic process from carbon monoxide, GO:2001134) (Rother and Metcalf, 2004; Lessner et al., 2006).

***Anaerobic biodegradation of biomass in animal intestines***. Foregut fermenting animals such as the ruminants (cattle, sheep, goats) as well as hindgut fermenters such as human, termites, and horse, employ variations of the overall process shown in Figure 2 for deriving nutrients from feed or food (Wolin, 1981; Wolin and Miller, 1983; Zinder, 1993; Miller and Wolin, 1996; Weimer, 1998; Hook et al., 2010; Sahakian et al., 2010). In cattle and many other foregut fermenters, the rumen serves as the first site for the degradation of forage (Wolin, 1981; Weimer, 1998; Hook et al., 2010). The residence time for the feed in rumen is rather short (5.6 h for the fluid and 35 h from particulates in rumen; compared to about 4.5 months even for nitrate, a soluble compound, in freshwater sediment) (Hristov et al., 2003), which is not conducible for significant growth and activity of slow-growing fatty acid-fermenting bacteria and acetoclastic methanogens (Zinder, 1993). Thus, in this digestive chamber the fatty acids and acetate are not converted to methane, rather are absorbed by the animal for nutrition (Zinder, 1993). The hydrogen and formate produced during the fermentation are converted to methane by methanogenic archaea. All plant material contains pectin, a methylated carbohydrate, and leaves, shoots and fruit are particularly rich in it. Anaerobic degradation of pectin (anaerobic pectin catabolic process, GO:1990489) serves as an important source of methanol in anaerobic environments (Schink et al., 1981). Thus, ruminants could carry methanogens in their rumens capable of utilizing methanol for methanogenesis and in some cases this has been shown to be true (Mukhopadhyay et al., 1992; Zinder, 1993).

In the hindgut of humans, i.e., the large intestine, the undigested material delivered from the small intestine is fermented, generating fatty acids, some hydrogen, and formate, and the latter two are converted to methane (Wolin, 1981; Zinder, 1993; Miller and Wolin, 1996; Sahakian et al., 2010; Flint, 2011). The process is beneficial to the host as it provides the fatty acids as additional nutrients. However, an uncontrolled production of fatty acids in this hindgut activity has been identified as one of the possible causes of obesity (Schmitz and Langmann, 2006; Nakamura et al., 2010; Sahakian et al., 2010; Flint, 2011).

In certain foregut fermenters such as kangaroos and wallabies and hindgut fermenters such as termites, the removal of hydrogen during biodegradation of complex polymers occurs through acetate formation and not methanogenesis (Brune and Friedrich, 2000; Gagen et al., 2010; Klieve et al., 2012).

#### Anaerobic biomass degradation in engineered systems: waste treatment and methane production from renewable resources

Aerobic treatment of municipal and industrial wastes via methods such as activated sludge requires energy input for supplying oxygen (Switzenbaum, 1983; Zinder, 1993). The process also generates a significant amount of microbial biomass (Zinder, 1993), which cannot be discharged to waterways (Zinder, 1993; Paul and Debellefontaine, 2007). In contrast, anaerobic methods not only require much less energy input and produce very little microbial biomass, but also conserve most of the energy present in the waste materials in the form of methane, which can be recovered as fuel (Zinder, 1993; Gao et al., 2014). Anaerobic waste treatment and the production of methane as biofuel from renewable resources follow the basic biological process (macromolecules catabolic process, GO:0009057) that has been described above for methanogenic biodegradation of biomass in sediments (Zinder, 1993). The mixture of methane and carbon dioxide that is produced in all these cases is known as biogas (Ducom et al., 2009). The biogas obtained from waste treatment facilities as well as from bio-digesters processing high sulfur feedstock contains a substantial amount of hydrogen sulfide and nitrogen oxides (Zinder, 1993; Fdz-Polanco et al., 2001; Janssen et al., 2001; Ducom et al., 2009; Diaz and Fdz-Polanco, 2012). Several methods for the removal of these unwanted compounds have been developed and research for developing even better separation methods continues (Fdz-Polanco et al., 2001; Janssen et al., 2001; Ducom et al., 2009; Diaz and Fdz-Polanco, 2012).

### Methanogenesis

**Figure 4:**
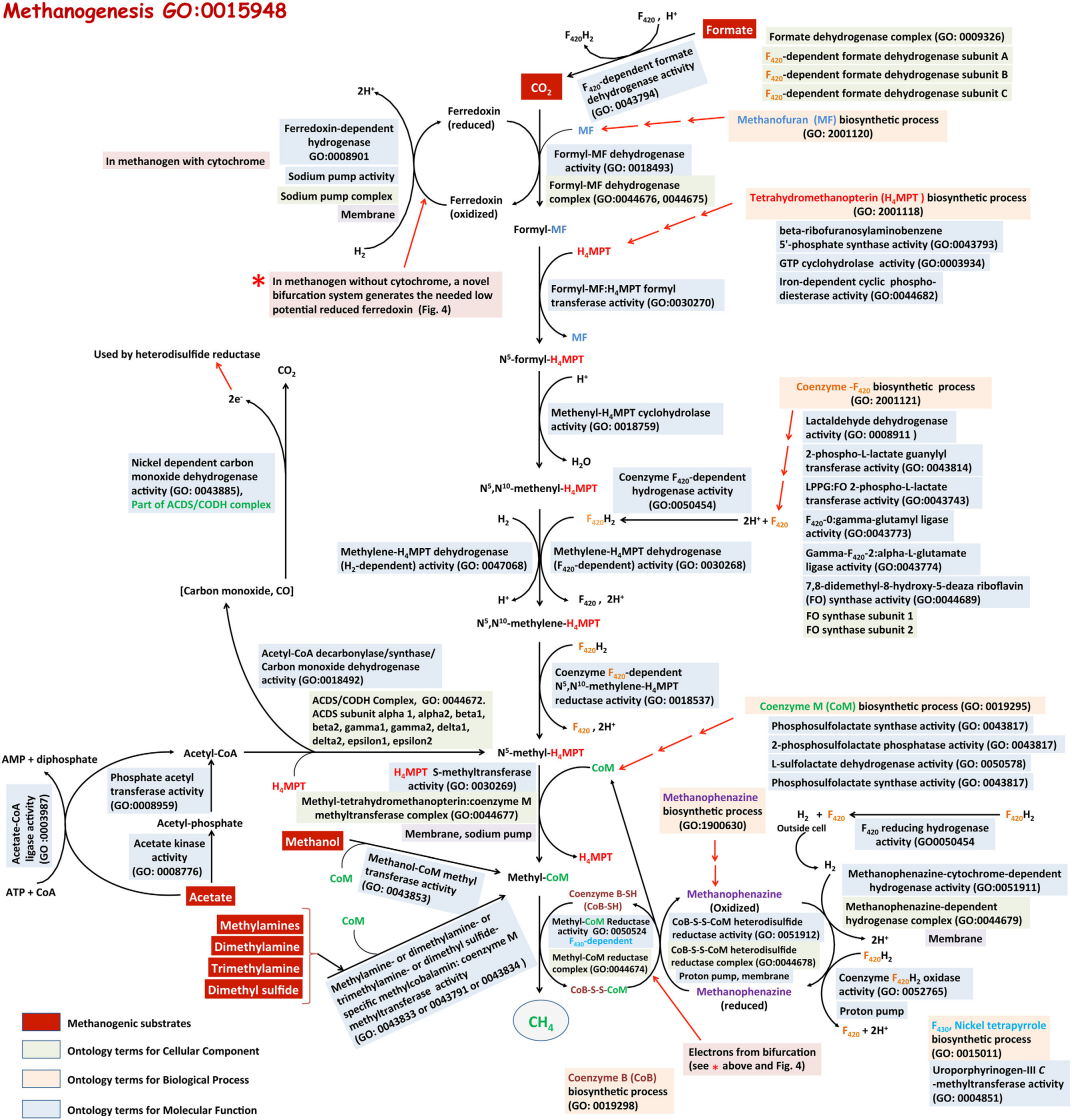
**Methanogenesis from various substrates, and relevant GO terms**. Methanogenic substrates (shaded in red) are biologically converted to methane by methanogenic archaea. This pathway requires several unique coenzymes. Biosynthesis processes of these coenzymes are also described in GO (shaded in beige). Color codes for three ontologies: Biological Process (shaded in light orange), Molecular Function (shaded in blue) and Cellular Component (shaded in green). The relevant references are in the text. This is a modified version of a figure available at our MENGO project website: http://www.mengo.biochem.vt.edu/pathways/bio_synthetic_pathways.php.

The pathways for methanogenesis or methane biosynthetic process (GO: 0015948) from various substrates and the respective molecular functions are shown in Figure 4. Here, the steps leading from CO_2_ to CH_4_ form the core, which is used in part or its entirety with other substrates as well (Wolfe, 1991, 1993; Ferry, 1993, 1999, 2011; Thauer et al., 1993, 2008; Deppenmeier et al., 1999; Deppenmeier, 2002; Deppenmeier and Muller, 2008; Liu and Whitman, 2008). These pathways utilize several unusual coenzymes of which methanofuran (MF), tetrahydromethanopterin (H_4_MPT), tetrahydrosarcinapterin (H_4_SPT), and coenzyme M (or HS-CoM) carry the carbon moiety destined to generate methane, while coenzyme F_420_ (a deazaflavin derivative), coenzyme B (HS-CoB or HS-HTP), methanophenazine, and coenzyme F_430_ (a tetrapyrrole) transfer electrons that are used in carbon reduction (Wolfe, 1991, 1993; Ferry, 1999; Deppenmeier and Muller, 2008; Thauer et al., 2008). Many unique enzymes and unusual mechanisms are also involved (Wolfe, 1991, 1993; Ferry, 1999; Deppenmeier and Muller, 2008; Thauer et al., 2008). In the following narrative the term H_4_MPT represents both H_4_MPT and H_4_SPT, which serve the same function in different organisms.

#### Methanogenesis from H_2_ plus CO_2_

This process (GO:0019386) utilizes hydrogen as the primary source of electron or reductant (Ferry, 1999; Deppenmeier and Muller, 2008; Thauer et al., 2008). It can operate with or without the involvement of cytochromes (Ferry, 1999; Deppenmeier and Muller, 2008; Thauer et al., 2008). The latter is utilized by methanogens that lack cytochromes (Ferry, 1999; Deppenmeier and Muller, 2008; Thauer et al., 2008) and is considered one of the most ancient respiratory metabolisms on earth (Leigh, 2002). We describe the process starting with the carbon transfer and reduction steps, followed by the energy production avenues.

***Carbon transfer and reduction***. It is believed that carbon dioxide (CO_2_) is captured by methanofuran (MF) to form an unstable compound called carboxy-MF (Thauer et al., 1993) which is reduced by formyl-MF dehydrogenase (GO:0018493) in an energy-dependent (endergonic) manner to formyl-MF with a low-potential ferredoxin (Fd) serving as electron carrier (Thauer et al., 2008). Formyl-MF dehydrogenase exists in two forms, one of which contains molybdenum (Fmd) and the other tungsten (Fwd) (Thauer et al., 1993); molybdenum and tungsten are found to be bound to a molybdopterin and growth conditions dictate which metal will be incorporated (Hochheimer et al., 1995). At the next step the formyl group is transferred to H_4_MPT by a transferase enzyme (Ftr, GO:0030270) to form formyl-H_4_MPT (Donnelly and Wolfe, 1986; Breitung and Thauer, 1990; Thauer et al., 1993). From this stage H_4_MPT carries four forms of the fixed carbon representing three oxidation states (Wolfe, 1991, 1993; Thauer et al., 1993, 2008; Ferry, 1999; Deppenmeier and Muller, 2008). First formyl-H_4_MPT is dehydrated by methenyl-H_4_MPT cyclohydrolase (Mch, GO:0018759) to form methenyl-H_4_MPT (Donnelly et al., 1985; Dimarco et al., 1986; Mukhopadhyay and Daniels, 1989; Klein et al., 1993), which in turn is reduced to methylene-H_4_MPT by the action of one of the two enzymes, F_420_-dependent methylene-H_4_MPT dehydrogenase (Mtd, GO:0030268) and a Fe-containing hydrogenase (Hmd, GO:0047068) (Hartzell et al., 1985; Mukhopadhyay and Daniels, 1989; Von Bunau et al., 1991; Schworer et al., 1993; Thauer et al., 1993; Mukhopadhyay et al., 1995). Mtd utilizes reduced F_420_ (F_420_H_2_) as reductant whereas Hmd retrieves electrons from molecular hydrogen (H_2_) (Hartzell et al., 1985; Mukhopadhyay and Daniels, 1989; Von Bunau et al., 1991; Schworer et al., 1993; Thauer et al., 1993; Mukhopadhyay et al., 1995). Methanogens with Hmd also carry paralogs of this protein (HmdII and HmdIII), but these proteins do not reduce methylene-H_4_MPT (Lie et al., 2013). Two roles of HmdII and HmdIII have been proposed: a. guiding the maturation of Hmd and b. linking energy production and protein synthesis (Oza et al., 2012; Lie et al., 2013). Methylene-H_4_MPT is reduced with F_420_H_2_ and by the action of F_420_-dependent methylene-H_4_MPT reductase (Mer, GO:0018537), providing the last H_4_MPT derivative on the pathway, methyl-H_4_MPT (Ma and Thauer, 1990; Te Brommelstroet et al., 1990; Ma et al., 1991; Thauer et al., 2008). The transfer of the methyl group from methyl-H_4_MPT to coenzyme M is catalyzed by a membrane-bound sodium ion (Na^+^)-pumping enzyme complex called methyl-H_4_MPT:coenzyme M methyl transferase (Mtr, GO:0044677) (Becher et al., 1992; Kengen et al., 1992; Gartner et al., 1993). This complex not only yields methyl-coenzyme M (CH_3_-CoM), but also generates a Na^+^-gradient that is used for energy production (see below) (Ferry, 1999; Deppenmeier and Muller, 2008; Thauer et al., 2008). The next step in the sequence yields methane. This last carbon-reduction reaction is catalyzed by CH_3_-CoM reductase (GO:0044674) with coenzyme B (HS-CoB or HS-HTP) serving as an electron source, resulting in a heterodisulfide, CoM-S-S-CoB, as product in addition to methane (Wolfe, 1991, 1992; Ferry, 1999; Deppenmeier and Muller, 2008; Thauer et al., 2008). The heterodisulfide is reduced by a reductase (Hdr, GO:0051912) to regenerate HS-CoM and HS-CoB (Ferry, 1999; Deppenmeier and Muller, 2008; Thauer et al., 2008). Hydrogen-oxidizing methanogens often carry two CH_3_-CoM reductase isozymes (McrI and McrII) (Rospert et al., 1990), one of which is effective under high hydrogen availability and the other under low hydrogen conditions (Rospert et al., 1990).

***Energy conservation***. First, we describe the details for methanogens lacking cytochromes. The first site of energy conservation is the Mtr reaction (Ferry, 1999; Deppenmeier and Muller, 2008; Thauer et al., 2008). The Na^+^-gradient generated at this step is directly used for the production of ATP by a membrane-bound AoA1-ATP synthase (GO:1990490) (Deppenmeier and Muller, 2008). Under certain conditions this gradient assists two membrane-associated and energy-converting hydrogenase complexes, EhaA-T and EhbA-Q, to generate reduced Fd with the ability to deliver low redox potential electrons (Thauer et al., 2008; Costa et al., 2010; Lie et al., 2012). The reduced Fd molecules generated by EhaA-T are used for the endergonic formyl-MF dehydrogenase reaction that yields formyl-MF, and those provided by EhbA-Q are used for cellular biosynthesis (Porat et al., 2006; Thauer et al., 2008; Costa et al., 2010; Major et al., 2010; Kaster et al., 2011; Lie et al., 2012). The next energy yielding step is the reduction of CH_3_-CoM and it is not clear whether the methanogens conserve this energy or the energy is released to strongly favor the forward reaction toward methane formation (Thauer et al., 2008). The reduction of CoM-S-S-CoB involves rather complex electron transfer mechanisms and also is a major site for energy conservation (Thauer et al., 2008; Costa and Leigh, 2014).

**Figure 5:**
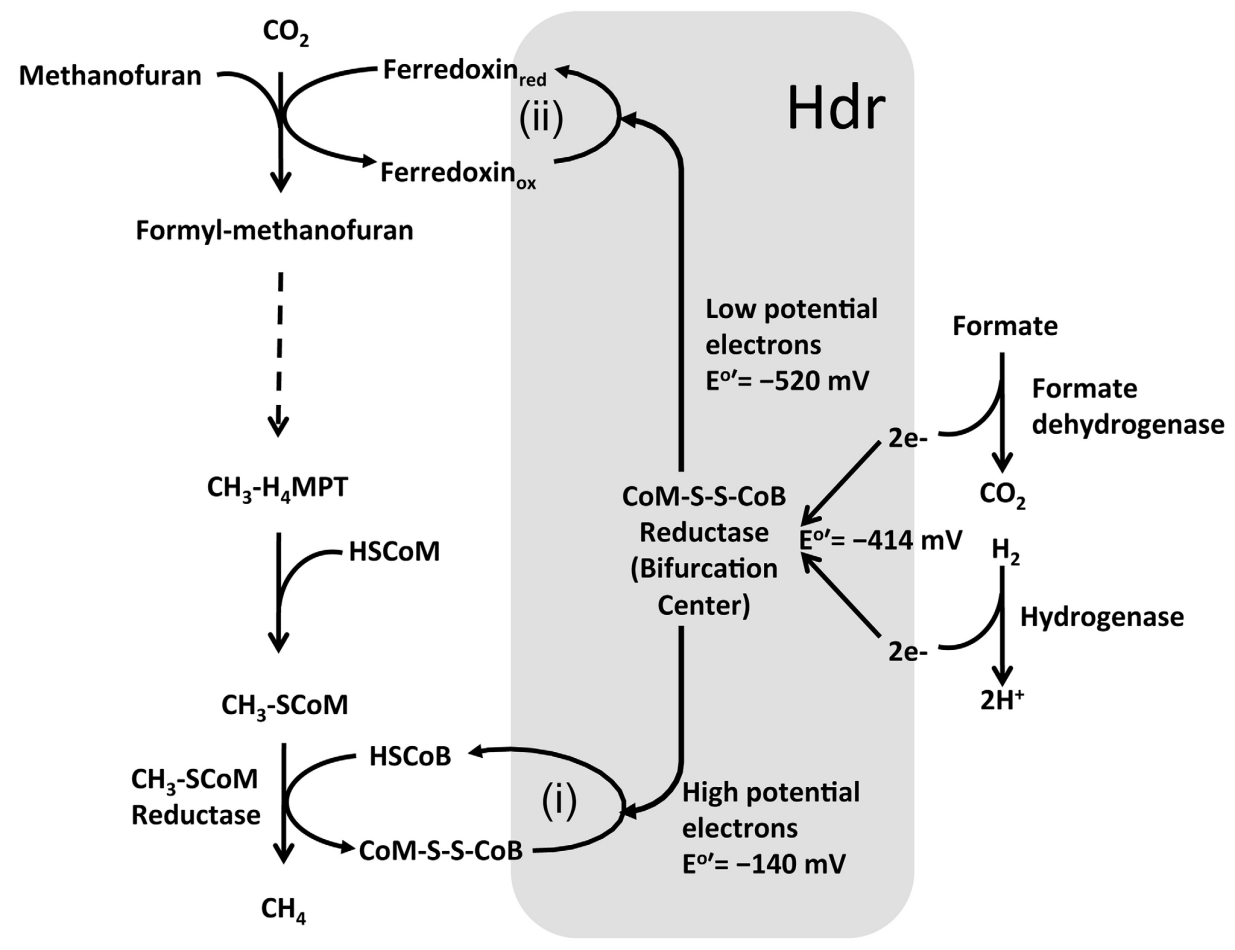
**Electron bifurcation in Methanogenesis**. HS-CoB or HS-HTP, coenzyme B; HS-CoM, coenzyme M; CoM-S-S-CoB, heterodisulfide of coenzyme M and coenzyme B. Heterodisulfide reductase (Hdr) utilizes bifurcated energy electrons for two purposes: (i) converting CoM-S-S-CoB to HS-CoM and HS-CoB, using high potential electrons; and (ii) reducing a low potential ferredoxin using low potential electrons, which is energetically suitable for the highly endergonic reduction of CO_2_ and generation of formyl-MF (Thauer et al., 2008; Thauer, 2012; Costa and Leigh, 2014).

In certain methanogens without cytochromes, the reduction of CoM-S-S-CoB and formyl-MF generation is coupled via a novel mechanism called bifurcation (Thauer et al., 2008; Costa et al., 2010; Kaster et al., 2011; Lie et al., 2012) (Figure 5). Here, the Vhu hydrogenase retrieves electrons from hydrogen and transfers those to soluble heterodisulfide reductase (Hdr). Hdr utilizes these electrons for two purposes (Figure 5): (i) converting CoM-S-S-CoB to HS-CoM and HS-CoB, which requires a relatively lower investment of energy; and (ii) reducing a low potential ferredoxin, which is energetically suitable for the highly energy intensive reduction of CO_2_ and generation of formyl-MF (Thauer et al., 2008; Costa et al., 2010; Kaster et al., 2011; Lie et al., 2012). This novel mechanism, where a single input (electrons of moderately low potential) is used to generate two outputs (two pools of electrons, with potentials that are higher and much lower than the input) is called electron bifurcation (GO:MENGO-UR). It is a major factor in energy conservation in methanogens as it helps to perform a highly endergonic reaction, such as the generation of formyl-MF, without an investment of ATP or an ion gradient (Thauer et al., 2008; Costa et al., 2010; Kaster et al., 2011; Lie et al., 2012). It seems to be an important tool for energy poor anaerobes (Thauer et al., 2008; Kaster et al., 2011). When withdrawal of intermediates from the methanogenesis pathway for biosynthesis causes a drop in CoM-S-S-CoB levels, the bifurcation process is rendered less efficient (Lie et al., 2012); in that case, as described in the preceding paragraph, an ion-driven hydrogenase system (EhaA-T) is employed for the generation of formyl-MF and this could be considered to be a type of anaplerosis (GO:MENGO-UR) (Lie et al., 2012).

Methanogens with cytochromes do not employ the bifurcation mechanism. Instead, a membrane-bound complex composed of a cytochrome-containing heterodisulfide reductase (HdrDE) (GO:0044678) and a hydrogenase (VhoECG) where VhoC is a b-type cytochrome is utilized (Thauer et al., 2008). The electrons derived from hydrogen by VhoECG are utilized by HdrDE for reduction of CoM-S-S-CoB (Thauer et al., 2008). The overall process is exergonic and thus, in addition to reducing CoM-S-S-CoB, the VhoECG-Hdr complex utilizes excess energy to extrude protons out of the cell. The low potential reduced ferredoxin, which is needed for the generation of formyl-MF, is provided by a proton-gradient-driven membrane-bound energy-converting hydrogenase complex (EchA-F).

As mentioned above, several methanogenesis enzymes form large protein complexes (GO:0043234) and some these are membrane bound (GO:0019898) and include specialized non-enzyme units such as ion pumps and lipid soluble small compounds. One example is the soluble heterodisulfide reductase complexes of methanogens that lack cytochromes, and can be described using a molecular function term, GO:0051912 (“CoB-CoM heterodisulfide reductase activity”) and two cellular component terms GO:0044678 (“CoB-CoM heterodisulfide reductase complex”) and GO:0043234 (“protein complex”). In the case of cytochrome-containing methanogens, one further additional cellular component GO term is available for a full description, namely GO:0019898 (“extrinsic component of membrane”).

#### Methanogenesis from formate

The carbon transfer and reduction steps in this process (GO:2001127) are similar to those described above for methanogensis from H_2_+CO_2_ (GO: 0015948). Both the CO_2_ and reducing power are derived from formate by the action of an F_420_-dependent formate dehydrogenase (FdhABC) (GO:0043794) (Schauer and Ferry, 1982; Lie et al., 2012); FdhAB subunits form the enzyme that produces CO_2_ and reduced F_420_ or F_420_H_2_ (HCOO^−^ + H^+^ + F_420_ → CO_2_ + F_420_H_2_) and FdhC is thought to import formate into the cell (Wood et al., 2003). CO_2_ is converted to methane using the CO_2_-reduction pathway described in Figure 4. Some of the reduced F_420_ (F_420_H_2_) participates directly in the Mtd and Mer reactions and a part of it is used by a bifurcating complex that provides electrons of appropriate redox potentials to heterodisulfide reductase and formyl-MF dehydrogenase. In the composition and some of the properties this bifurcating complex differ from the one employed for methanogenesis from H_2_+CO_2_ (see above). When a methanogen grows on formate, a part of the Fdh pool associates with the Hdr and Vhu/Vhc hydrogenases, and together with a formyl-MF dehydrogenase they form a bifurcating complex (Lie et al., 2012). This Fdh-containing bifurcating complex utilizes electrons from F_420_H_2_ (produced by Fdh) and generates high and low potential electrons, either directly or via production of hydrogen as intermediate, that are consumed in the reduction of CoM-S-S-CoB and the generation of formyl-MF, respectively (Lie et al., 2012).

#### Methanogenesis from ethanol or secondary alcohols plus carbon dioxide

Only a few methanogens can perform methanogenesis with secondary alcohol as electron source (GO:MENGO-UR; also secondary alcohol catabolic process, GO:MENGO-UR) (Widdel, 1986; Bleicher et al., 1989; Widdel and Wolfe, 1989; Schirmack et al., 2014). These substrates are oxidized to their respective ketones to provide reducing equivalents for the reduction of carbon dioxide to methane via the pathway shown in Figure 3 (Boone et al., 1993; Zinder, 1993). Ethanol, when used, is converted to acetaldehyde (methanogenesis with ethanol as electron source, GO:MENGO-UR; ethanol catabolic process, GO:0006068). These conversions are consistent with the general observation that methanogens cannot break carbon-carbon bonds in energy substrates other than that found in acetate (see below). Two types of alcohol dehydrogenase have been found in these organisms: one reduces nicotinamides (NAD^+^ or NADP^+^) (GO:0004022 and GO:0008106), and the other transfers electrons to coenzyme F_420_ during alcohol oxidation (GO:0052753) (Widdel and Wolfe, 1989). Most of these enzymes have broad specificities allowing the organisms to use ethanol, 2-propanol, 2-butanol, 2-pentanol, cyclopentanol, cyclohexanol, and 2,3-butanediol (Bleicher et al., 1989).

#### Methanogenesis from carbon monoxide

Many methanogens can utilize carbon monoxide (CO) although higher levels of this gas inhibit growth of these archaea (Daniels et al., 1977; O'brien et al., 1984; Rother and Metcalf, 2004; Lessner et al., 2006). Three routes of CO utilization have been found in these organisms. In one, called methanogenesis from carbon monoxide (GO:2001134), CO is simply oxidized to CO_2_ by carbon monoxide dehydrogenase (CODH) (GO:0008805), and the resulting two electrons are used for either hydrogen production (GO:1902422) or ferredoxin reduction (Daniels et al., 1977; Ferry, 1999; Vepachedu and Ferry, 2012). Then the hydrogen and/or reduced ferredoxin are used for methanogenesis from CO_2_ (GO:0019386). Overall, for every four moles of CO oxidized, one mole of methane and 3 moles of CO_2_ are produced. The second mode of CO utilization has been found in *Methanosarcina acetivorans* where methanogenesis (GO: 0015948) is inhibited by CO but growth is not (Rother and Metcalf, 2004). This organism uses two non-methanogenic routes for energy production (Rother and Metcalf, 2004), the primary one being acetogenic (acetate biosynthetic process, GO:0019413) and the secondary one being formate-forming (formate biosynthetic process, GO:0015943). Even under these conditions methanogenesis operates at low rates, primarily to provide cellular biosynthetic precursors (Rother and Metcalf, 2004). Here methanogenesis from CO_2_ involves novel enzymes that transfer the methyl group of CH_3_-H_4_MPT to CH_3_-CoM and serve in the accompanying energy conservation (Lessner et al., 2006); the methyl transfer step could involve a cytoplasmic methyltransferase (CmtA) in addition to a membrane-bound methyl-H_4_MPT:coenzyme M methyl transferase (Mtr) (Vepachedu and Ferry, 2012). In the third route, CO promotes the production of dimethyl sulfide and methanethiol (3CO + H_2_S + H_2_O → CH_3_SH + 2CO_2_) and energy is conserved via a yet to be identified system (Moran et al., 2008).

#### Methanogenesis from methanol, methylamines and methanethiols

Methanogenesis from all of these substrates involves the formation of methyl-CoM as an intermediate (Ferry, 1999). When methanol serves as the sole substrate for methanogenesis (GO:0019387), it provides both carbon and reductant for methanogenesis and this process consumes four moles of methanol for every three moles methane generated. Of these, one mole of methanol is oxidized to CO_2_, generating six-electron equivalents of reductant, which are then used to convert three moles of methanol to three moles of methane (Keltjens and Vogels, 1993). The oxidation of methanol to CO_2_ involves a part of the CO_2_ reduction pathway, but in the reverse direction (Figure 3) (Wassenaar et al., 1998). The methyl groups enter this oxidation process at the methyl-coenzyme M stage by the action of two methyl transferases, MT1 and MT2 or MT2-M (Van Der Meijden et al., 1984a,b; Keltjens and Vogels, 1993; Wassenaar et al., 1998; Ferry, 1999). MT1 is a two-subunit enzyme (MtaBC) and MT2-M has one subunit (MtaA). The first reaction involves transfer of the methyl group of methanol by MT1 to the corrinoid co-factor of its MtaC subunit; this is an automethylation process (Van Der Meijden et al., 1984a,b; Wassenaar et al., 1998). Then MT2-M or MtaA transfers the methyl group from MtaC to HS-CoM, generating methyl-coenzyme M(Van Der Meijden et al., 1984a,b; Wassenaar et al., 1998). Existence of isozymes of MT1 catering to the growth on methanol under various conditions has also been reported (Bose et al., 2006). The methyl groups destined for oxidation are transferred from CH_3_-CoM to H_4_MPT by the membrane-bound methyl-H_4_MPT:coenzyme M methyl transferase (Mtr) (Fischer et al., 1992; Sauer et al., 1997; Ferry, 1999). This endergonic reaction is assisted by a Na^+^-gradient and generates CH_3_-H_4_MPT (Deppenmeier et al., 1999; Ferry, 1999; Deppenmeier and Muller, 2008). The steps from CH_3_-H_4_MPT to CO_2_ are a reversal of those used for CO_2_-reduction, except the organisms performing this process lack Hmd and F_420_-dependent Mtd performs the oxidation of methylene-H_4_MPT to methenyl-H_4_MPT (Thauer et al., 1993; Deppenmeier et al., 1999; Ferry, 1999; Deppenmeier and Muller, 2008).

Utilization of mono-, di- and tri-methylamines (MMA, DMA, and TMA) for methanogenesis (GO:2001128, GO:2001129, GO:2001130 respectively) follows the general process that is described above for methanol except that substrate-specific methyl transferases are involved in the transfer of methyl groups to coenzyme M. Using MT1 and MT2 of the methanol systems as the reference the methylamine-specific methyl transferases have been named as follows (Wassenaar et al., 1996, 1998; Ferguson and Krzycki, 1997; Burke et al., 1998; Ferry, 1999; Ferguson et al., 2000; Paul et al., 2000; Bose et al., 2008): for MMA, MMAMT+MMCP (MT1) and MT2-A (MT2); for DMA, DMA-MT (MT1) and MT2-A (MT2); for TMA, TMA-52+TCP (MT1) and MT2-A (MT2). For methanogenesis from TMA, MT2-A could be substituted by MT2-M (Ferry, 1999). Methanogenesis from methylated thiols (methanethiol, dimethylsulfide, or methylmercaptopropionate; GO:2001133, GO:2001131, and GO:2001132) also involves special methyl transferase proteins (Tallant et al., 2001; Bose et al., 2009). For example, dimethylsulfide is converted to methyl-CoM by the actions of MtsB (MT2) and MtsA (MT2) (Tallant et al., 2001).

Energy conservation during methanogenesis from methylated compounds occurs in at least two ways. The F_420_H_2_ generated during the oxidation of the methyl group of CH_3_-H_4_MPT to CO_2_ is oxidized via the membrane-bound F_420_H_2_-dehydrogenase complex (reduced coenzyme F_420_ dehydrogenase activity, GO:0043738), and in the process a lipid soluble membrane-resident cofactor called methanophenazine is reduced (Deppenmeier and Muller, 2008). These events lead to the extrusion of two protons per F_420_H_2_ oxidized (Deppenmeier and Muller, 2008). There is another avenue that produces the same outcome and it begins with the release of molecular hydrogen through the oxidation of F_420_H_2_ by a soluble F_420_-dependent hydrogenase (Frh, GO:0050454) (Kulkarni et al., 2009). This hydrogen upon its release from the cell is captured by a membrane-bound hydrogenase complex (Vht/Vtx) (GO:MENGO-UR, GO:MENGO-UR), which transfers electrons generated from the oxidation of hydrogen to methanophenazine and releases two protons outside the cell (Kulkarni et al., 2009). In certain methanogens the latter process is the major route of F_420_H_2_ oxidation (Kulkarni et al., 2009). The reduced methanophenazine produced by these reactions is utilized by the membrane-bound heterodisulfide reductase (Hdr)-cytochrome b2 complex (GO:MENGO-UR) for the reduction of CoM-S-S-HTP, and this process provides two more protons outside the cell (Deppenmeier and Muller, 2008; Kulkarni et al., 2009; Welte and Deppenmeier, 2014). All these extruded protons generate proton-motive force, which drives ATP synthesis (GO:0015986) via an ATP synthase (GO:0045259) (Deppenmeier and Muller, 2008; Kulkarni et al., 2009; Welte and Deppenmeier, 2014).

#### Methanogenesis from H_2_ plus methanol

A GO term for this process has recently been proposed by us (methanogenesis from H_2_ and methanol, GO:1990491). Here, the methyl group of methanol is transferred to coenzyme M by two methyl transferases, MT1 and MT2, producing methyl-CoM (Keltjens and Vogels, 1993). The rest of the process, the reduction of methyl-CoM by HS-CoB, the reduction of CoM-S-S-CoB by electrons derived from hydrogen, and the energy conservation, likely follows the system described in the section on methanogenesis from H_2_ plus CO_2_; an exception is *Methanosphaera stadtmanae*, which grows only on H_2_ plus methanol with a supplement of acetate (Miller and Wolin, 1985), as it would employ the cytochrome-independent system (Fricke et al., 2006).

#### Methanogenesis from acetate

About 70% of the biologically produced methane originates from acetate (GO:0019385) (Ferry, 1992, 1993, 1999). The methyl group of acetate is reduced to methane and the carboxyl group is oxidized to CO_2_ providing the reductant for methyl reduction (Ferry, 1992, 1993, 1999, 2011; Thauer et al., 2008). The process begins with the activation of acetate by the action of one of two systems, one involving acetate kinase and phosphotransacetylase (GO:0008776 and GO:0008959) and the other catalyzed by acetyl-CoA synthase (synonym of acetate-CoA ligase activity, GO:0003987), both generating acetyl-CoA (Aceti and Ferry, 1988; Jetten et al., 1989; Lundie and Ferry, 1989; Ferry, 1992, 1993, 1999, 2011; Thauer et al., 2008). The first route generates ADP that is converted back to ATP via electron transport phosphorylation at an ATPase (Ferry, 1992, 1993, 1999). In contrast, the second route generates AMP and pyrophosphate, and AMP has to be converted to ADP by adenylate kinase (GO:0004017) through the consumption of one ATP (AMP + ATP → 2ADP) before it can used for the regeneration of ATP (ADP + P_i_ + energy → ATP) (Jetten et al., 1989; Zinder, 1993; Berger et al., 2012). Thus, organisms utilizing the acetyl-CoA synthase reaction are placed in an energetically unfavorable situation and exhibit slow growth rates (Zinder, 1993). However, by virtue of this investment they are able to utilize acetate even at very low concentrations and consequently are the predominant acetotrophic methanogens in many anaerobic niches of nature (Zinder, 1993). It is not known whether the energy present in pyrophosphate is conserved or is released via hydrolysis for the purpose of making the acetate activation process thermodynamically more favorable (Welte and Deppenmeier, 2014). The methanogens employing acetyl-CoA synthase carry pyrophosphatase (GO:0016462) and whether the enzyme is positioned to harvest or release energy is not known (Berger et al., 2012; Welte and Deppenmeier, 2014). The next step, the breakage of the carbon-carbon bond of the acetate moiety in acetyl-CoA, is catalyzed by an acetyl-CoA decarbonylase/synthase-carbon monoxide dehydrogenase complex (GO:0044672) (Ferry, 1993, 1999; Lu et al., 1994; Grahame, 2003; Li et al., 2006; Wang et al., 2011). The carbonyl group of acetyl-CoA is oxidized to CO_2_ by the CODH component (GO:0043885) and the reducing equivalents (two-electrons) generated by this process help to reduce ferredoxin (Ferry, 1993, 1999, 2011; Lu et al., 1994; Grahame, 2003; Li et al., 2006; Wang et al., 2011). The methyl group of the acetyl group is transferred to H_4_MPT via a corrinoid cofactor of the CODH/ACDS complex, producing CH_3_-H_4_MPT (Ferry, 1999; Grahame, 2003). The methyl group of CH_3_-H_4_MPT leads to methane via the actions of methyl-H_4_MPT:coenzyme M methyl transferase (Mtr) and methyl-CoM reductase (Figure 4). The CO_2_ produced from acetate is hydrated to bicarbonate by a membrane-bound gamma-type carbonic anhydrase (GO:0004089) and is efficiently exported out of the cell (Ferry, 2011). This process is thought to improve the thermodynamic efficiency of methanogenesis from acetate (Ferry, 2011).

There are two avenues for energy conservation in methanogenesis from acetate (Deppenmeier and Muller, 2008). One is via the use of the sodium potential generated by Mtr and has been described above. The other is through the oxidation of reduced ferredoxin through one of two complex processes. Certain acetotrophic methanogens oxidize reduced ferredoxin by use of Ech hydrogenase, generating molecular hydrogen and proton potential (Meuer et al., 1999, 2002; Kulkarni et al., 2009). The molecular hydrogen is utilized for the extrusion of additional protons and for heterodisulfide reduction via the Vho hydrogenase, methanophenazine and heterodisulfide reductase, as during methylotrophic methanogenesis (Figure 4; see above) (Kulkarni et al., 2009). In methanogens lacking Ech hydrogenase, a complex called Rnf utilizes reduced ferredoxin, producing a sodium gradient and transferring electrons to heterodisulfide reductase via methanophenazine. Thus, Rnf is considered a replacement of the Ech and Vho hydrogenases (Li et al., 2006; Wang et al., 2011). Both the H^+^ and Na^+^ potentials are utilized by an A_1_A_O_ ATP synthase (GO:1990490) for ATP production (Deppenmeier and Muller, 2008); in some cases a Na^+^/H^+^ antiporter (GO:0015385) called Mrp adjusts the ratio of the two gradients for optimizing the thermodynamic efficiency of the ATP synthase (Li et al., 2006; Wang et al., 2011; Jasso-Chavez et al., 2013).

#### Biosynthesis of methanogenesis coenzymes

Many of the coenzymes involved in methanogenesis, namely methanofuran, tetrahydromethanopterin, tetrahydrosarcinapterin, coenzyme M, coenzyme F_420_, coenzyme B, methanophenazine, and coenzyme F_430_, have unusual properties. As a result, the respective biosynthesis pathways have attracted attention (Graham and White, 2002). This interest has increased further as some of these coenzymes have been found to perform critical functions in other organisms, such as in actinobacteria (includes mycobacteria and streptomyces groups), methanotrophic and methylotrophic bacteria, cyanobacteria, and plants (Takao et al., 1989; Batschauer, 1993; Purwantini et al., 1997; Chistoserdova et al., 2004; Krishnakumar et al., 2008). Some of the existing knowledge has been summarized at the MENGO website.

## Synthetic biology exploitation of methanogenesis pathways in methanogens

Exploitation of methanogens for the production of methane from unnatural substrates has begun. For example, *Methanosarcina acetivorans* has been made proficient in converting methyl acetate to methane and carbon dioxide, and in converting methyl propionate to methane and propionate. This was achieved by expressing a broad-specificity esterase (hydrolase activity, acting on ester bonds, GO:0016788) from *Pseudomonas veronii* in *M. acetivorans* (Lessner et al., 2010). Wild type *M. acetivorans* exhibits only a minor esterase activity. The heterologous esterase in the engineered strain releases methanol from these two esters, and methanol is used for methanogenesis following the pathways described above. Acetate, the other product from methyl acetate is also converted to methane whereas propionate generated from methyl propionate is excreted (Lessner et al., 2010).

## A new route for biological production of methane

A recent discovery (Metcalf et al., 2012; Yu et al., 2013) shows that some of the abundant marine archaea and bacteria, which are distinct from the well-known methanogenic archaea, are likely major producers of methane in nature. Methane is abundant in the oceans, but the source was unclear (Reeburgh, 2007). Methylphosphonate was suspected as the source as genes encoding carbon-phosphorus lyases are common in marine microbes, but the biosynthetic pathway for methylphosphonate was unknown (Karl et al., 2008). It has recently been shown that the marine archaeon *Nitrosopumilus maritimus* encodes a pathway for methylphosphonate biosynthesis and it produces cell-associated methylphosphonate esters (Metcalf et al., 2012). The production of methylphosphonate seems to be a widespread process in marine microorganisms, and that when facing phosphorus-limitation these organisms would degrade methylphosphonate to obtain phosphorus, thus releasing methane (Metcalf et al., 2012). The GO database lacks description for methylphosphonate biosynthetic and catabolic processes, as well for the following key enzymes: carbon-phosphorus (C-P) lyase, producer of methane from methylphosphonate; phosphonoacetaldehyde dehydrogenase (Pdh) and methylphosphonate synthase (MPn), two key enzymes for methylphosphonate biosynthesis (Metcalf et al., 2012). However, the terms for the first two enzymes on the methylphosphonate biosynthesis pathway that starts from phosphoenolpyruvate, namely “phosphoenolpyruvate mutase (Ppm) activity” (GO:0050188) and “phosphonopyruvate decarboxylase (Ppd) activity,” do exist (GO:0033980). To cover this new biological process for methane production we have proposed the following new GO terms: phosphonate carbon-phosphorus lyase activity (GO-MENGO-UR); “methane biosynthetic process” (GO:0015948), a parent term; two child terms, “aerobic methane biosynthetic process” (GO:MENGO-UR) and “anaerobic methane biosynthetic process” (GO:MENGO-UR).

## GO annotation

To begin the application of the GO terms to annotating genomes of methanogenic microbes, we have performed GO annotation of the relevant gene products encoded by these genomes. The annotations we created were based solely on experimental evidence (e.g., results from direct assays or mutant phenotypes), in order to provide “gold standards” for subsequent machine annotations. These annotations are available at the MENGO website under the Gene Annotations menu (Gene Annotations for Natural Biological System; Gene Annotations for Synthetic Biological System). Forms for the submission of new annotations (Submit New Gene Annotation for Natural or Synthetic Biological System) are available under the same menu.

**Figure 6:**
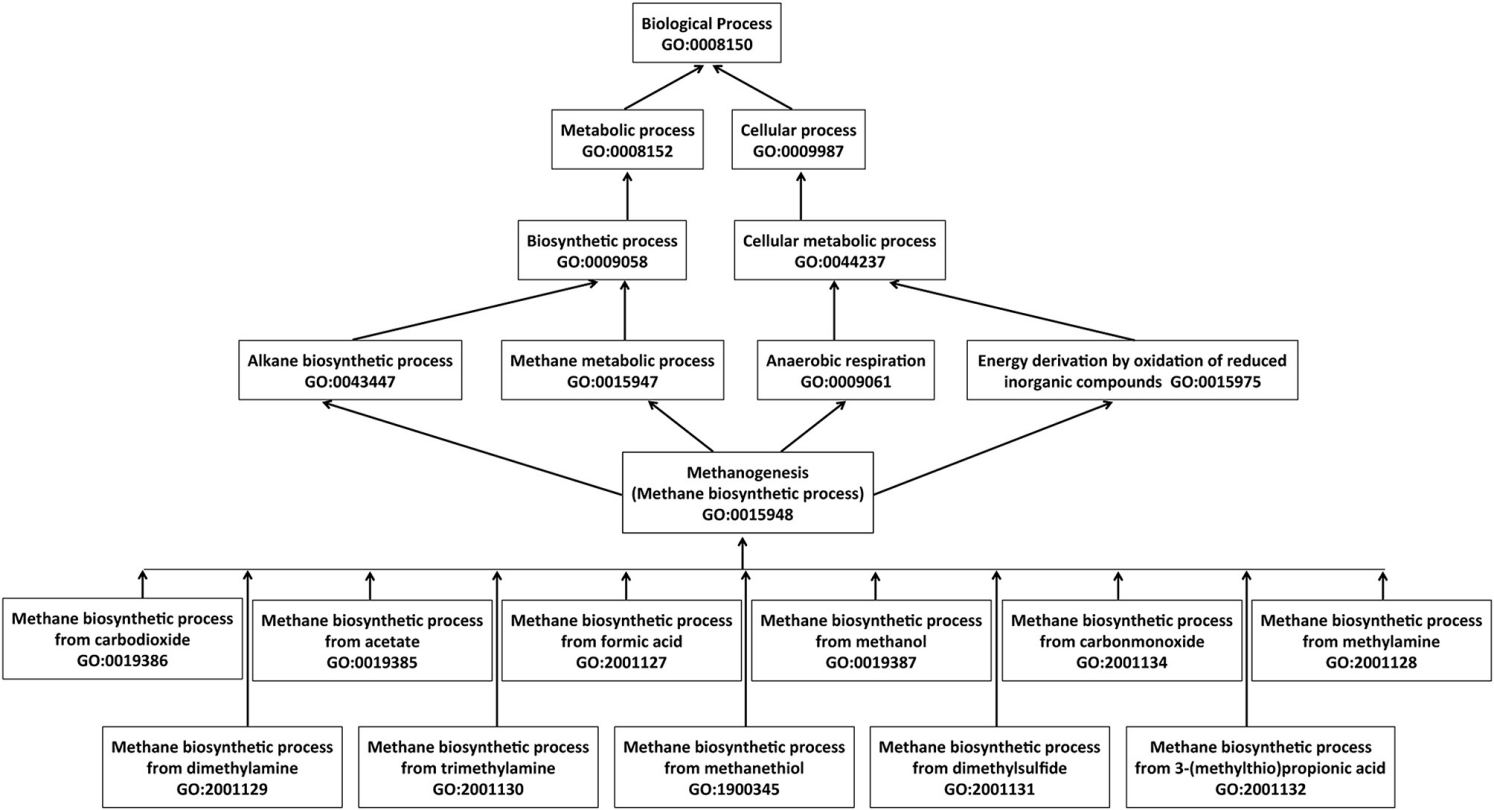
**Simplified graphical view of hierarchical terms for methane biosynthetic process in the Gene Ontology**. The arrows represent “is_a” relationship, where an arrow begins at a child term and points to a parent term. Multiple child terms for a parent term as well multiple parent terms for a child term exist.

We have annotated 80 gene products with the parent term “methane biosynthetic process” (GO: 0015948) along with appropriate child terms (Figure 6). These genes were categorized into three groups; 51 gene products for methanogenesis pathways, 19 gene products for biosynthesis of coenzymes specifically used in methanogenesis, and 10 genes for coenzyme metabolism (see Table S1, Supplementary material).

## Concluding remarks

The goal of the MENGO project is to develop a set of GO terms for describing gene products involved in energy-related microbial processes. GO allows annotations of gene products using terms from three ontologies: molecular function, biological process, and cellular component. The GO embodies structured relationships among the terms and the annotations provide links between gene products and the terms (Figure 6). This combination allows researchers to infer possible functional roles of gene products in diverse organisms. A set of relevant gene products well-annotated with GO terms will assist bioenergy researchers to efficiently design synthetic biological systems for commercially viable biofuel production, as it will allow effective mining for optimal parts from a larger natural inventory. For example, one could mine for amenable parts of a methanogenesis system from all available genomes, including those of organisms that do not produce methane. Thus, the GO terms and associated “gold standard” manual annotations that the MENGO has developed should provide the foundation for a growing resource that is of wide value to the microbial bioenergy community. We encourage members of the research community to participate in our effort toward the development of additional GO terms and performing manual annotations of gene products with potentials of application in bioenergy production and bioremediation. The MENGO website provides electronic forms for the submission of candidate GO terms and annotations for review and subsequent submission to the GO database.

### Conflict of interest statement

The authors declare that the research was conducted in the absence of any commercial or financial relationships that could be construed as a potential conflict of interest.

## Abbreviations

CODH/ACDS: acetyl-CoA decarbonylase/synthase-carbon monoxide dehydrogenase complex
F_420_: coenzyme F_420_ or 7,8-didemethyl-8-hydroxy-5-deazaflavin derivative
F_430_: coenzyme F_430_ - a tetrapyrrole
GO:MENGO-UR: GO terms generated in the MENGO project, submitted to the GO consortium and awaiting acceptance
HS-CoM: coenzyme M
HS-CoB or HS-HTP: coenzyme B
H_4_MPT: tetrahydromethanopterin
H_4_SPT: tetrahydrosarcinapterin
MF: methanofuran.
